# An Innovative Protocol for Metaproteomic Analyses of Microbial Pathogens in Cystic Fibrosis Sputum

**DOI:** 10.3389/fcimb.2021.724569

**Published:** 2021-08-27

**Authors:** Alexander C. Graf, Johanna Striesow, Jan Pané-Farré, Thomas Sura, Martina Wurster, Michael Lalk, Dietmar H. Pieper, Dörte Becher, Barbara C. Kahl, Katharina Riedel

**Affiliations:** ^1^Institute of Microbiology, Department of Microbial Physiology & Molecular Biology, University of Greifswald, Greifswald, Germany; ^2^Research Group ZIK Plasmatis, Leibniz Institute for Plasma Science and Technology, Greifswald, Germany; ^3^Center for Synthetic Microbiology, Department of Chemistry, Philipps-University Marburg, Marburg, Germany; ^4^Institute of Microbiology, Department of Microbial Proteomics, University of Greifswald, Greifswald, Germany; ^5^Institute of Biochemistry, Department of Cellular Biochemistry & Metabolomics, University of Greifswald, Greifswald, Germany; ^6^Research Group Microbial Interactions and Processes, Helmholtz Centre for Infection Research, Braunschweig, Germany; ^7^Institute of Medical Microbiology, University Hospital Münster, Münster, Germany

**Keywords:** cystic fibrosis, sputum, microbial community, microbiome, 16S sequencing, metaproteomics, metabolomics, *in vivo*

## Abstract

Hallmarks of cystic fibrosis (CF) are increased viscosity of mucus and impaired mucociliary clearance within the airways due to mutations of the cystic fibrosis conductance regulator gene. This facilitates the colonization of the lung by microbial pathogens and the concomitant establishment of chronic infections leading to tissue damage, reduced lung function, and decreased life expectancy. Although the interplay between key CF pathogens plays a major role during disease progression, the pathophysiology of the microbial community in CF lungs remains poorly understood. Particular challenges in the analysis of the microbial population present in CF sputum is (I) the inhomogeneous, viscous, and slimy consistence of CF sputum, and (II) the high number of human proteins masking comparably low abundant microbial proteins. To address these challenges, we used 21 CF sputum samples to develop a reliable, reproducible and widely applicable protocol for sputum processing, microbial enrichment, cell disruption, protein extraction and subsequent metaproteomic analyses. As a proof of concept, we selected three sputum samples for detailed metaproteome analyses and complemented and validated metaproteome data by 16S sequencing, metabolomic as well as microscopic analyses. Applying our protocol, the number of bacterial proteins/protein groups increased from 199-425 to 392-868 in enriched samples compared to nonenriched controls. These early microbial metaproteome data suggest that the arginine deiminase pathway and multiple proteases and peptidases identified from various bacterial genera could so far be underappreciated in their contribution to the CF pathophysiology. By providing a standardized and effective protocol for sputum processing and microbial enrichment, our study represents an important basis for future studies investigating the physiology of microbial pathogens in CF *in vivo* – an important prerequisite for the development of novel antimicrobial therapies to combat chronic recurrent airway infection in CF.

## Introduction

Cystic fibrosis is the most common inherited monogenic disorder in Caucasian populations with an incidence of approx. one in 3,000 births (O’Sullivan and Freedman, 2009). The disease is caused by mutations in the cystic fibrosis transmembrane conductance regulator (CFTR) gene, encoding an anion channel localized in epithelial cells e.g. of the respiratory and gastrointestinal tract (Chmiel and Davis, 2003). More than 1,500 mutations of the CFTR gene are described, which all lead to the CF phenotype. Most importantly, the CF phenotype is characterized by an impaired ion homeostasis, which, in consequence, leads to a sticky, dehydrated mucus within the respiratory tract and an impaired mucociliary clearance (Ratjen, 2009). Ultimately, these hallmarks of CF pave the way for the colonization by opportunistic microbial pathogens establishing chronic infections, which starts already early after birth, and is considered to be the main reason for mortality (Rogers et al., 2014). Typically, *Staphylococcus aureus* and *Haemophilus influenzae* represent early colonizers, which are followed by other bacterial pathogens including e.g. *Pseudomonas aeruginosa*, *Burkholderia cepacia* complex and *Stenotrophomonas maltophilia* but also fungal pathogens like *Aspergillus fumigatus* and *Candida albicans*, and viruses (e.g. influenza and respiratory syncytial virus) (Filkins and O'Toole, 2015). The polymicrobial communities within the CF lung are highly dynamic and differ greatly from patient to patient. In the past few years, culture independent diagnostic methods revealed even larger diversity of core genera, which are abundant in the majority of adult patients including *Streptococcus* and *Neisseria*, as well as obligate anaerobes like *Prevotella*, *Veillonella*, and *Catonella* (Rogers et al., 2014; Filkins et al., 2015).

Of note, CF airways are characterized by an inflammatory milieu, which can be attributed to the microbial colonization/infection eliciting a host immune response characterized by the dysregulation of epithelial innate immunity and airway leukocytes. Proteolytic and oxidative products derived from an exuberant immune response in combination with microbial virulence factors are the main reasons for lung tissue damage, which ultimately lead to respiratory failure and death (Cohen and Prince, 2012; Eiserich et al., 2012; Kamath et al., 2015). Thus, deeper insights into these complex polymicrobial infections, focusing on the (patho-)physiology of the microbial CF lung community as well as host-microbe interactions are of essential importance for a better understanding of the disease progression and the development of novel treatment strategies.

In the past, (meta-)proteomics approaches were used as a powerful tool to investigate the physiological alterations of lung tissues and body fluids (e.g. bronchoalveolar lavage, blood, feces, and sputum) in CF patients as well as CFTR post-translational modifications and CF biomarkers (Eiserich et al., 2012; Kamath et al., 2015; Debyser et al., 2016; Liessi et al., 2020). However, most of these studies were limited by focusing on the host perspective while overlooking the microbial side of infection. Studies characterizing the bacterial and fungal pathogens of CF lungs were typically performed *in vitro*, using lung isolates grown under lung-mimicking conditions (Kamath et al., 2015). Consequently, novel approaches for the *in vivo* analyses of the microbial pathophysiology directly at the site of infection are urgently needed. Here, we present the first *in vivo* microbial metaproteome analysis, complemented by 16S sequencing, metabolomics, and microscopic analyses to study microbial communities and facultative microbial pathogens within CF sputum. To this end, we established an innovative sputum processing protocol, which overcomes major technical and analytical challenges of CF sputum including (I) limited sample volume, (II) challenging processability of CF sputum due to its viscous and slimy character, (III) extraction of nucleic acids, proteins and metabolites out of a single sputum sample, (IV) enormous dominance of human proteins (e.g. mucins, albumins, immunoglobulins) over microbial proteins of interest, and (V) high abundance of (neutrophil-derived) proteases unspecifically digesting microbial proteins of interest (Kamath et al., 2015). In this protocol, a combination of differential centrifugation and filtration is used as key elements for the enrichment of bacterial cells significantly increasing bacterial protein identification coverage.

Our study represents a fundamental basis for follow-up studies investigating the microbial metaproteome and bacterial pathophysiology in CF sputum, which is an essential prerequisite for the development of innovative antimicrobial treatment approaches.

## Experimental Procedures

### Study Cohort, Ethics Statement, and Sputum Sampling

In total, 24 sputum samples derived from 20 different patients were collected. The study was approved by the institutional ethics review board Münster, Germany (2010-155-f-S). Of the 24 sputum samples, 21 were used as test samples to establish a reliable, reproducible and widely applicable protocol suitable for sputum processing, nucleic acid extraction, microbial enrichment and subsequent protein and metabolite extraction. As a proof of concept, three samples, which were derived from three individual patients (designated Patient A, Patient B, and Patient C, respectively) were selected for detailed 16S sequencing, metaproteome and metabolome analyses. Clinical data of these three patients are summarized in **Table 1**. Patient B carried the homozygous Phe508del CFTR genotype, while Patients A and C carried other CFTR-mutations. Importantly, antibiotic therapy of all three patients finished before the time point of sputum sample collection, reducing the risk of false functional analysis due to lysed and/or dead microbial cells. The three Patients A, B, and C were selected based on their differences in age, lung function, antibiotic therapy, disease progression, and microbial lung community structure in order to show the applicability of our sputum processing protocol over preferably diverse samples.

**Table 1:** Clinical Data of the three CF patients A, B, and C included in metaproteome and metabolome analyses.

	Patient A	Patient B	Patient C
Age	19	24	38
Sex	male	male	male
Exacerbation acc. to Fuchs^a^	0	0	1
FEV1% predicted^b^	81%	52%	28%
Antibiotic therapy	Cefaclor	Cefuroxim	Amoxicillin/Clavulanic acid, Meropenem
CFU/mL *S. aureus*	1.4 x 10^7^	3.6 x 10^6^	3.2 x 10^7^
CFU/mL *P. aeruginosa*	–	–	1.5 x 10^8^
Quantification Neutrophiles^c^	3	2	2
Quantification Epithelial Cells^c^	1	2	2

a 0 = no exacerbation; 1 = a minimum of 4 criteria acc. to Fuchs pertain (Fuchs et al., 1994).

b FEV = forced expiratory volume at 1 s.

c 1 = 1 cell/field of view, 2 = up to 10 cells/field of view, 3 = up to 100 cells/field of view.

The freshly expectorated sputum samples were immediately chilled on ice and transported to the laboratory for further processing. Next, samples were transferred into 5 mL reaction tubes, three ceramic beads (diameter approx. 2 mm) were added and the samples were homogenized using a Retsch mill at 15 Hz for 120 s (Stokell et al., 2014). Aliquots of the homogenized sputum samples were stained according to the Gram procedure and numbers of neutrophils, epithelial cells and bacteria were semi-quantitatively evaluated according to standard diagnostic procedures for CF specimens (Gilligan, 2014). Samples were aliquoted and diluted using 250 µL of homogenized samples and 250 µL ice-cold 0.9% NaCl. Glycerol was added to a final concentration of 10% and samples were subsequently stored at -80 °C for further analyses.

### Community Composition Analysis by 16S Sequencing

Samples were gently mixed with an equivalent volume of Sputolysin (10%) and incubated for 30 min at 37°C on a ThermoMixer. RNA was extracted using the RNeasy kit (Qiagen, Hilden, Germany) following the manufacturer’s instructions, but including a mechanical lysis step (Schulz et al., 2018). After DNA digestion, first-strand complementary DNA was synthesized using the Superscript IV First-Strand Synthesis System (Invitrogen, Carlsbad, CA) and random primers, following the manufacturer’s instructions. DNA was extracted from the samples using the FastDNA Spin Kit for Soil (MP Biomedicals, Solon, OH, USA) following the manufacturer’s instructions (Camarinha-Silva et al., 2014). Amplicon libraries covering the V1-V2 region of the 16S rRNA gene were amplified in a two-step PCR as previously described (Rath et al., 2017) and sequenced on a MiSeq (2X250 bp, Illumina, Hayward, California, USA). Bioinformatic processing was performed as previously described. Raw reads were merged with the Ribosomal Database Project (RDP) assembler (Cole et al., 2013). Sequences were aligned within MOTHUR (gotoh algorithm using the SILVA reference database) and subjected to preclustering (diffs=2) (Schloss et al., 2009) yielding so-called phylotypes that were filtered for an average abundance of ≥0.001% and a sequence length ≥250 bp before analysis. Phylotypes were assigned to a taxonomic affiliation based on the naïve Bayesian classification (Wang et al., 2007) with a pseudo-bootstrap threshold of 80%. Phylotypes were then manually analyzed against the RDP database using the Seqmatch function. A species name was assigned to a phylotype when only 16S rRNA gene fragments of previously described isolates of that species showed a seqmatch score >0.95.

### Sputum Sample Processing and Microbial Enrichment

**Figure 1:**
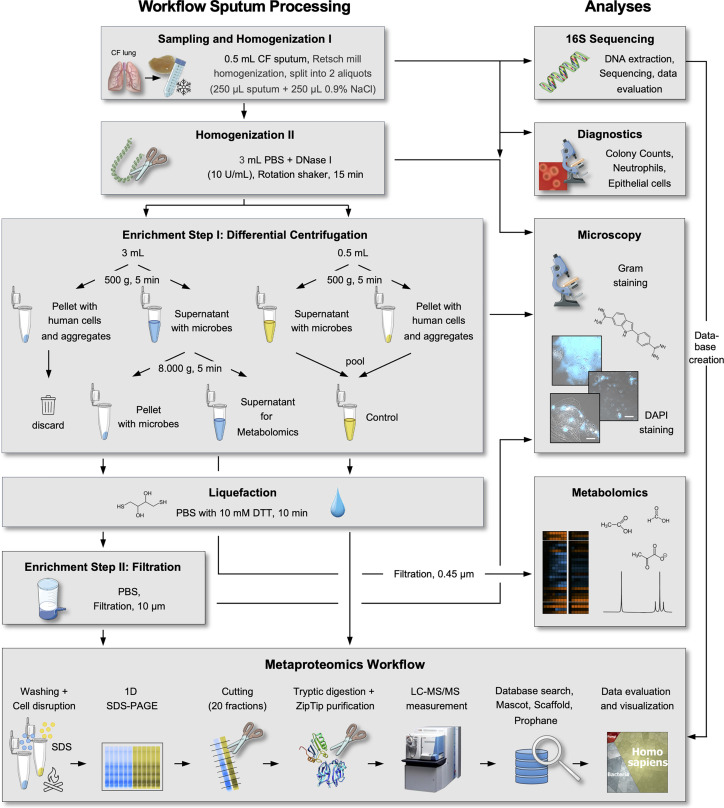
Workflow of sputum sample processing. Methodological details for microbial enrichment, 16S sequencing, metaproteome, metabolome, and microscopic analyses are schematically depicted.

All sputum processing steps were carried out at 4 °C in order to minimize changes of the *in vivo* sputum metaproteome and the metabolome, respectively. The entire workflow is summarized in **Figure 1**. Homogenization of 500 µL sputum samples (250 µL Retsch mill treated sputum plus 250 µL 0.9% NaCl) was performed by adding 3 mL ice-cold PBS_EDTA/PIC_ (137 mM NaCl, 0.2 mM KCl, 10 mM Na_2_HPO4, 1.8 mM KH_2_PO4, pH 7.4, plus 10 mM EDTA, and 1 tablet protease inhibitor cocktail (PIC, cOmplete, Mini, Sigma-Aldrich) per 10 mL), which was additionally supplemented with DNase I (10 U/mL, ThermoFisher) to break down eDNA-based aggregates (Shak et al., 1990). The samples were subsequently incubated on a rotation shaker (Stuart, Cole-Parmer) at 20 rpm for 15 min. Success of the further homogenization and breakdown of eDNA-based aggregates was microscopically verified (see below). The homogenized sputum suspensions (3.5 mL) were split into a first sub-sample (3 mL) for further enrichment of microbial cells and a second sub-sample (500 µL) to obtain a non-enriched control.

For enrichment of microbial cells, the first sub-sample was subjected to differential centrifugation as the first enrichment step of microbial cells. Here, the samples were centrifuged at 500 g for 5 min (keeping cell lysis as low as possible) and the pellet containing human cells and bigger aggregates was discarded. The supernatant, which contained microbial cells, was subsequently centrifuged at 8.000 g for 5 min. The resulting supernatant was filter-sterilized (0.45 µm cut-off, Sarstedt) and used for metabolome analyses (see below). The pellet was resuspended in 500 µL PBS_EDTA/PIC_, which was additionally supplemented with DTT (10 mM, Sigma-Aldrich) and incubated at 4 °C for 10 min on a rotation shaker (Stuart, Cole-Parmer) to further homogenize and liquify the sample. As the second enrichment step for microbial cells, the cell suspension was subsequently filtered (10 µm cut-off, Merck) to remove remaining human cells/aggregates, the filter was washed with 1 mL ice-cold PBS_EDTA/PIC_ and the filtrate was collected. The filtrate containing enriched microbial cells was then centrifuged (8,000 g, 5 min), and the pellet was washed twice using ice-cold TE_PIC_-buffer (10 mM Tris-HCl, 1 mM EDTA, pH 8, containing 1 tablet protease inhibitor cocktail (PIC, cOmplete, Mini, Sigma-Aldrich) per 10 mL) to further reduce contamination by human proteins. The washed pellet was resuspended in 200 µL TE_PIC_ and subjected to protein extraction for MS-analyses as described below.

In order to keep preparation protocols of the enriched sample and the non-enriched control as similar as possible, the non-enriched control sample was also subjected to differential centrifugation as described above (500 g, 5 min followed by 8,000 g, 5 min), pooled again and also subjected to liquefaction using 500 µL PBS_EDTA/PIC_ with DTT (10 mM, Sigma-Aldrich). The suspension was incubated and again centrifuged as described above. The pellets were resuspended using 200 µL TE_PIC_, pooled again, and subjected to protein extraction for MS-analyses as described below.

### Protein Extraction

Suspensions of enriched microbial cells and the non-enriched control were subjected to mechanical cell disruption as previously described (Becher et al., 2009; Zühlke et al., 2016) since this method was shown to effectively disrupt one of the most robust cell types we expected in our samples – the gram-positive, spherical cocci of *S. aureus*. Due to limited sputum-sample volume and the concomitant small number of microbial cells in the enriched sample, the cell disruption described by (Becher et al., 2009; Zühlke et al., 2016) was downscaled using 200 µL of the respective suspensions and 150 mg glass beads (0.1 to 0.11 mm, Sartorius Stedim Biotech) in 0.5 mL cryotubes (Sarstedt) followed by 3 homogenization cycles at 6.5 m/s for 30 s with intermitted cooling on ice for 1 min in a FastPrep-24™ classic bead beating grinder and lysis system (MP Biomedicals). Subsequently, the samples were centrifuged (15,000 g, 4°C, 5 min) and the tube content (including glass beads and cell debris) was transferred into a fresh 1.5 mL reaction tube. 200 µL 2x extraction buffer (100 mM Tris-HCl, 0.3 M NaCl, 2 mM EDTA, 4% SDS, pH 8.5, adapted from (Chourey et al., 2010) were added and the suspension was boiled at 95°C and 1.200 rpm for 10 min in a thermo-shaker (Eppendorf). Glass beads and cell debris were pelleted by centrifugation at 15,000 g at 4°C for 5 min and the supernatant, representing the protein extract, was collected. Proteins were concentrated (approx. 4-fold) in a vacuum centrifuge (Eppendorf AG) for 1 h followed by determination of the protein concentration using the BCA-assay microplate procedure (ThermoFisher) according to the manufacturer’s instructions.

### MS Sample Preparation

40 µg protein per sample were mixed 3:1 with an SDS-sample buffer (15% glycerol, 5% 2-mercaptoethanol, 2.4% SDS, 0.8% Tris, 0.005% bromophenol blue), boiled for 10 min at 95°C and subsequently separated on a 4-12 % SDS-polyacrylamide gradient gel (Criterion, BioRad). The gel was fixed, washed and stained using Colloidal Coomassie Brilliant Blue G-250 as previously described (Laemmli, 1970; Neuhoff et al., 1988). After the staining procedure, excessive Coomassie stain was removed from the gel using water. Subsequently, gel lanes were fractionated into 20 gel pieces, cut into gel blocks of approx. 1 mm^3^ and prepared for MS/MS analysis as described by (Lassek et al., 2015). Obtained peptides were resolved in 0.1% acetic acid and desalted using ZipTips (C18, Merck Millipore). The desalted peptide mixtures were again vacuum-dried and stored at -80°C until MS/MS analysis.

### MS/MS Analysis

Purified peptides were reconstituted with 0.1% acetic acid and analyzed by reversed phase liquid chromatography (LC) electrospray ionization (ESI) MS/MS using an Orbitrap Elite mass spectrometer (Thermo Fisher Scientific, Waltham, USA). Nano-reversed-phase-LC columns (20 cm length x 100 µm diameter) packed with 3.0 µm C18 particles (Dr. Maisch GmbH, Ammerbuch-Entringen, Germany) and heated to 45°C were used to separate the purified peptides with an EASY-nLC 1200 system (Thermo Fisher Scientific, Waltham, USA). The peptides were loaded with solvent A [0.1% acetic acid (v/v)] and subsequently eluted by a non-linear gradient from 2% to 99% solvent B (0.1% acetic acid (v/v), 95% acetonitrile) at a flow rate of 300 nl*min^-1^ over 91 min. A full scan was recorded in the Orbitrap with a resolution of 60,000 at m/z 400. The twenty most abundant precursor ions were consecutively isolated and fragmented *via* collision-induced dissociation (CID) with a normalized collision energy of 35. Singly charged ions and ions with unassigned charge state were rejected and lock mass correction as well as dynamic exclusion (fragmented precursors were excluded from fragmentation for 30 s) were enabled. Each sample was measured twice, creating two technical replicates per sample.

### Metaproteomics Data Base Assembly and Search

Three patient-specific databases were constructed based on the phylogenetic information derived from community composition analysis by 16S sequencing. In order to keep the databases and concomitant computational costs as small as possible, genera with a relative abundance of less than 0.1% according to sequencing results were not considered (**Table S1**). The following protein sequences were added: *Homo sapiens*, the most common (pathogenic) fungal genera in CF (*Aspergillus, Blumeria, Candida, Cladosporium, Cryptococcus, Exophiala, Rasamsonia, Rhodotorula, Saccharomyces, Scedosporium*, and *Sporobolomyces* according to (Chotirmall and McElvaney, 2014; Williams et al., 2016), common laboratory contaminants, and DNase I. For this purpose, FASTA protein sequences were downloaded from UniProt on September 18, 2018 and redundant entries were removed using the Linux-implemented FASTA tool kit resulting in three patient-specific protein databases, which contained 5.546.037 (Patient A), 4.024.158 (Patient B), and 3.331.936 (Patient C) entries, respectively. Database search was performed using the Mascot software (version 2.6.2, Matrix Science, Boston, MA, USA) with the following settings: peptide tolerance of 10 ppm, MS/MS tolerance of 0.8 Da, up to two missed cleavages allowed, methionine oxidation set as a variable modification, and carbamidomethylation set as a fixed modification. A second database search was performed using Scaffold (version 4.8.7, Proteome Software, Portland, OR, USA) and the built-in X! Tandem search engine with the same settings as described above, as well as the following settings: protein probability = 95%, peptide probability = 99%, single peptide identifications allowed. Here, Mascot and Scaffold used the given databases (containing bacterial, fungal, and human protein sequences) for an *in silico* digestion calculating theoretical peptide sequences and creating theoretical spectra thereof. These theoretical spectra were then matched with experimentally achieved MS/MS spectra for protein identification (Schiebenhoefer et al., 2020). Protein quantification was based on normalized spectral abundance factors (NSAF) as previously described (Zybailov et al., 2006; Zhu et al., 2010). Taxonomic and functional assignment of identified protein groups was performed using Prophane (Schiebenhoefer et al., 2020) (version 3.1.4) with the settings stated in **Table S2**. Here, Prophane provides an automated bioinformatic platform enabling the taxonomic and functional annotation of metaproteome data by integrating various databases (e.g. NCBI, Eggnog, Pfams) and algorithms (e.g. diamond blastp, Hmmr) (Schiebenhoefer et al., 2020).

### Metaproteomics Data Analyses and Visualization

Prophane output files were used to calculate the mean NSAF of both technical replicates and to create Voronoi treemaps (Bernhardt et al., 2009; Liebermeister et al., 2014) using the Paver software (version 2.1, DECODON GmbH, Greifswald, Germany). Here, Voronoi treemaps visualize taxonomic and functional diversity of sputum samples, respectively, according to relative NSAF-based abundances of different taxonomic genera. To this end, mean NSAF values of all protein/protein group were used, which were identified in at least one out of two technical replicates. Moreover, mean values of both technical replicates were used to visualize protein abundances in bar graphs according to NSAF-based relative abundance as well as absolute number of protein groups for each patient. The protein abundances of each patient were used to calculate mean values and to assess statistically significant differences of protein abundances between enriched and control samples by multiple unpaired t-tests. Enrichment factors of bacterial proteins/protein groups were calculated using three complementary approaches: (i) sum of all NSAFs in the enriched sample divided by the sum of all NSAFs in the control, (ii) absolute number of proteins/protein groups identified in the enriched sample divided by absolute number of proteins/protein groups identified in the control, (iii) percentage of proteins/protein groups identified in the enriched sample divided by absolute number of proteins/protein groups identified in the control.

### Metabolome Analyses

Samples were lyophilized overnight (Christ, Germany). Dried samples were derivatized for 90 min at 37°C in 40 µl Methoxyamine hydrochloride (MeOX) (20 mg/ml in pyridine) and afterwards for 30 min with 80 µl of N-methyl-N-(trimetylsilyl)trifluoroacetamide (MSTFA) at 37°C. Analytical GC-MS system consisted of an Agilent Technologies 7890B gas chromatograph and a mass selective detector (5977B Inert Plus Turbo MSD, Agilent Technologies). Injection was done with SSL (split/splitless) injector (G4513A, Agilent Technologies) (split 1:25 at 250°C, 1.0 μl; carrier gas: Helium with a flow of 1.0 ml/min). The MS operated in the electron impact mode with an ionization energy of 70 eV. The oven program started with 1 min at 70°C, was increased up to 76°C with 1.5°C/min followed by heating up to 220°C with 5°C/min and heating up to 325°C with 20°C/min. The final temperature of 325°C was hold for 8 min. Mass spectra were acquired in scan mode from 50-500 m/z at a rate of 2.74 scans/s and with a solvent delay of 6.0 minutes. Chromatography was performed using a 30 m HP-5 column (Agilent Technologies) with 0.25 mm i.d. and 0.25 μm film thickness. The detected compounds were identified by processing of the raw GC-MS data with MassHunter software Qual B.08.00 and comparing with NIST 2017 and Fiehn mass spectral databases and with retention times and mass spectra of standard compounds (inhouse database). The supplemented list contains compounds with scores to libraries of 70 or more (**Table S6**). Metabolites were relatively quantified among the three patient samples and depicted as circles, which areas correlate with metabolite abundance.

### Microscopic Analyses

25 µL of the samples derived from different sputum processing steps as indicated in **Figure 1** (after homogenization, after the first differential centrifugation step, after filtration) were transferred into a 96-well microtiter plate and diluted to an OD of 5 using PBS. OD was measured at 500 nm in a microtiter plate reader (Synergy MX, BioTek Instruments, Winooski, USA). Samples were stained in the dark at room temperature for 15 min using DAPI (2 µg/mL final concentration, Merck Millipore). 4 µL of these samples were applied on a thin layer of 1.5 % agarose in 0.9 % NaCl, which was mounted on a microscope slide. Phase contrast and fluorescence microscopy images were acquired and processed using a Zeiss Imager M2 (Carl Zeiss, Jena, Germany) equipped with a 100x/NA 1.3 oil immersion objective, a filter for monitoring DAPI fluorescence (excitation at 358 nm, emission at 461 nm), and the ZEN 2011 software package (Carl Zeiss, Jena, Germany). The number of human cells, particles/aggregates of different sizes, and microbial cells were counted in 50 randomly selected fields-of-view per sample. The results were averaged among Patient A, B, and C, and statistically significant differences were assessed by multiple unpaired t-tests.

## Results and Discussion

It is well described that microbial pathogens frequently establish infections in the airways of CF patients during infancy, which may become chronic, cause severe tissue damage and ultimately lead to death due to respiratory failure (Lyczak et al., 2002) - the leading cause of CF mortality (Rogers et al., 2014). However, the molecular mechanisms underlying co-infection, microbial interplay, and disease progression are still poorly understood.

In order to address these critical open questions, we developed an *in vivo* approach with a specific focus on the metaproteomic analyses of the CF microbiome, driven by 16S sequencing community composition analyses. We complemented these results by metametabolomic data acquired by metabolic footprint analyses, and microscopic data. Since major technical challenges, related to sputum consistency and processability, have so far precluded such analyses, we first established a reliable, reproducible and widely applicable protocol for sputum sample processing and subsequent metaproteomic and metabolomic analyses with a focus on microbial pathogens.

### A Metaproteomic and Metabolomic Analyses Protocol Overcoming Major Technical Challenges Related to CF Sputum Processing

We established a straight-forward workflow allowing nucleic acid extraction, metabolome footprint analyses and microbial protein enrichment and analyses from a single CF sputum sample. The major technical challenges and the different steps of our protocol addressing these technical challenges (**Figure 1**) are presented and discussed in the following paragraphs:

#### Limited Sample Volume

The amount of CF sputum sampled varies from patient to patient and rarely exceeds volumes of a few milliliters, which limits the biomass available for simultaneous nucleic acid, protein, and metabolite extraction. For this study, we collected 24 sputum samples derived from 20 different patients with a sample volume ranging from 0.3 ml to 2 ml (average = 0.7 ml, median = 0.6 ml). In order to establish a protocol, which is applicable to a great variety of different CF patients, we used a sputum volume of 0.5 ml as starting material. This amount was sufficient for simultaneous analyses of nucleic acids, proteins, and metabolites from a single sample.

#### Homogenization and Digestion of eDNA-Based Aggregates

**Figure 2:**
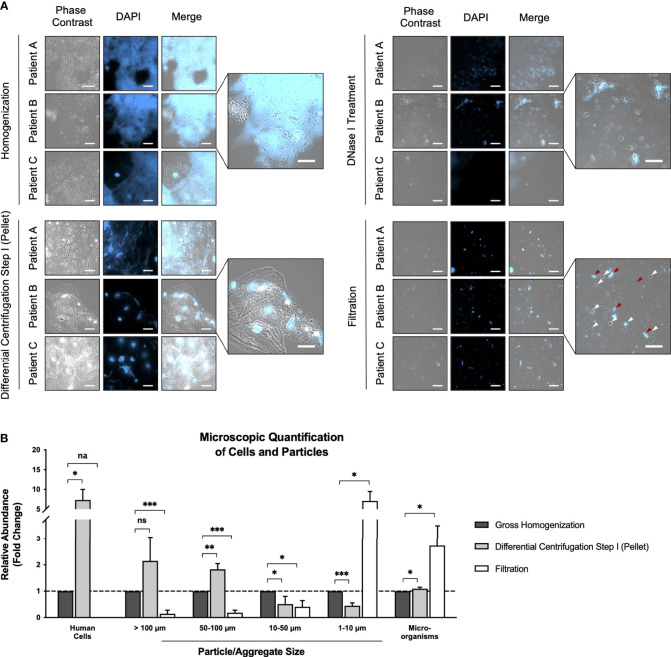
Microscopic analyses during microbial enrichment procedure. **(A)** Representative images of all three samples selected for metaproteome and metabolome analyses acquired after gross homogenization, DNase I treatment, differential centrifugation step I (pellet), and filtration as phase contrast, DAPI, and merged images. One merged image of each enrichment step is magnified for an improved visualization. White arrows indicate cocci, red arrows indicate rod-shaped bacteria. The scale bar represents 10 µm. **(B)** Quantification of human cells, particles/aggregates of varying size, and microorganisms in 50 images acquired in randomly selected fields of view after each enrichment step. Results are depicted as relative abundance (mean fold changes of all three sputum samples) ± standard deviation compared to quantitative values of the gross homogenization sample, which were set to 1. Statistical significance was assessed by multiple unpaired t-tests. Na, not applicable; ns, not significant; *p < 0.05, **p < 0.01, ***p < 0.001.

CF sputum represents a very viscous and slimy matrix due to macromolecules like eDNA and heavily glycosylated mucins (Stokell et al., 2014; Kamath et al., 2015), which complicates and prolongs downstream processing. Indeed, our microscopic analyses clearly showed massive cell clusters embedded in “clouds” of eDNA, which partially exceeded sizes of 500 µm. A common source of this eDNA are NETs (neutrophil extracellular traps): networks of primarily neutrophil-derived eDNA loaded with proteins, which show antimicrobial activity and simultaneously protect the eDNA from degradation (Herzog et al., 2019). To make cells trapped in these eDNA “clouds” accessible, they needed to be broken down prior to further processing (**Figure 2A**). Available techniques for sputum homogenization and liquefication include mechanical, chemical or enzymatic treatment at room temperature, or 37 °C (Palmer et al., 2007; Son et al., 2007; Fu et al., 2012; Yang et al., 2012; Wu et al., 2019). However, for metaproteome and metabolome analyses sputum processing needs to be carried out quickly and at 4 °C in order to avoid changes in the composition of the metaproteome and metabolome. Consequently, all sample processing steps were performed at 4 °C. We started our microbial enrichment protocol by homogenizing the sputum samples using a Retsch mill followed by the addition of ice-cold PBS including DNase I and subsequent incubation on a rotary shaker at 4°C. This combination of mechanical and enzymatic treatment resulted in a very efficient homogenization of the sputum samples. Following this treatment, samples can be pipetted easily and appear homogeneous with the naked eye. Fluorescence microscopy demonstrated that the aforementioned “clouds” of eDNA were successfully digested (**Figure 2A**). Importantly, DNase-treatment not only reduces viscosity of sputum samples (Shak et al., 1990), but also releases microbes trapped within eDNA “clouds” as we confirmed microscopically (**Figure 2A**). Furthermore, DNA digest also releases microbes from biofilms, which are frequently formed by microbial pathogens within the CF lung and contain eDNA as one of the major stabilizing components (Otto, 2008; Goerke and Wolz, 2010; Schwartbeck et al., 2016; Kovach et al., 2017). Thus, DNase-treatment at 4°C critically improves microbial enrichment and protein identification coverage of CF sputum samples.

Since sputum processing time needs to be kept as short as possible in order to preserve the metaproteome and the metabolome, we used sputum test samples to gradually reduce DNase I incubation time from 30 min to 10 min, without observing a decrease in DNA digestion efficiency (**Figure S1**), resulting in a total sputum processing time of approx. 60 min.

#### Avoiding Liquefaction and Homogenization Strategies Interfering With Metaproteome and Metabolome Analyses

Other methods for mechanical homogenization and eDNA breakdown like vortexing, intense shaking, and sonification, respectively, were intentionally avoided in order to keep human and microbial cells as much intact as possible, which is a prerequisite for unbiased metaproteome and metabolome quantification. Moreover, another commonly used method for sputum homogenization and liquefaction - the digestion of the sputum samples using proteases (Son et al., 2007; Wu et al., 2019) - was also avoided, because it would interfere with metaproteome analysis and would significantly decrease protein coverage. To further reduce unwanted protein degradation due to the high abundance of serine- and metalloproteases in CF sputum samples, a protease inhibitor cocktail was added to all processing steps for metaproteome preservation (Sloane et al., 2005; Kamath et al., 2015).

A further frequently applied strategy for sputum homogenization and liquefaction is the use of chemicals, primarily DTT (commercially available as Sputasol, Sputolysin, or Cleland’s reagent) (Stokell et al., 2014). Stokell et al. even considered DTT treatment mandatory, since it is not possible to pipette sputum samples due to their high viscosity without DTT treatment (Stokell et al., 2014). However, we avoided DTT treatment in early steps of our protocol, since high amounts of DTT inhibit DNase I activity and also interfere with metabolome analyses by masking other analytes. Therefore, DTT was only added at a late step of our protocol (after DNase-treatment and sampling aliquots for metabolome analysis) for liquefying the remaining pellet allowing filtration (**Figure 1**). However, depending on the viscosity of the remaining pellet, it should be carefully assessed, if DTT should be used or not since DTT can assist bacterial cell lysis and therefore might have a negative impact on microbial cell recovery (Liu et al., 2018).

#### Enrichment of Microbial Proteins Overcoming the Outnumbering Human Protein Abundance

The enormous abundance of human proteins (e.g. mucins, serine and metalloproteases, immunoglobulins, serum albumin) (Kamath et al., 2015) masks comparably low abundant microbial proteins during MS/MS analyses. Confirming this, we found high amounts of human proteins in the non-enriched controls (**Tables S3**–**S5**). In order not to increase this problem, we kept the first steps of sputum processing as mild as possible, to minimize lysis of human cells and a concomitant contamination of the microbial metaproteome (and metabolic footprint, see above). Thus, to further increase the amount of microbial proteins compared to human proteins, microbial cells were enriched, while human cells were depleted, prior to MS/MS analyses. Several approaches were tested using the sputum test samples to reduce sample complexity and enrich microbial cells: differential centrifugation (Tanca et al., 2014; Tanca et al., 2017), filtration (Xiong et al., 2015; Schultz et al., 2020), as well as density gradient centrifugation (Hevia et al., 2016). Each procedure was investigated for the enrichment success microscopically (**Figure S2**). Since *S. aureus* is one of the most prevalent and important CF pathogens, we additionally tested an enrichment protocol combining *S. aureus* specific antibodies and magnetic beads adapted from (Bicart-See et al., 2016; Wei et al., 2016) (**Figure S2**). However, neither density gradient centrifugation, nor antibody/magnetic bead enrichment resulted in a reproducible and sufficient enrichment. However, differential centrifugation as well as filtration did result in reproducible but only small enrichment of microbial cells as observed microscopically (data not shown). Therefore, differential centrifugation and filtration were combined resulting in a successful enrichment of microbial cells and an efficient depletion of human cells, respectively (**Figure 2**).

Although our enrichment strategy markedly increased the concentration of recovered microbial cells, a significant amount of biomass was lost during this two-step enrichment procedure. This reasons the necessity to use a higher starting volume of the homogenized sputum for microbial enrichment (3 mL) than for the non-enriched control (0.5 mL) ensuring that the protein yield after enrichment is still sufficiently high. Equal protein amounts (40 µg) of both the enriched samples and the non-enriched controls were used for metaproteome analyses to account for the different input volumes.

#### Increasing Protein Yield by Optimizing Cell Disruption and Protein Extraction

Moreover, it has been reported that the cell disruption method critically impacts the extraction efficiency and true species representation in various environmental samples (Starke et al., 2019). Therefore, the subsequent cell disruption and protein extraction process was optimized to increase protein yield. To this end, we used the sputum samples for protocol development and evaluated multiple cell disruption and protein extraction procedures. These included sonification, freeze and thaw cycles, boiling, bead beating, enzymatic treatment, harsh extraction buffers and different combinations of these procedures. Cell disruption and protein extraction efficiency was evaluated by measuring extracted protein concentrations and total protein amounts, respectively (data not shown). Based on these results, we decided to use a combination of downscaled beat-beating adapted from (Becher et al., 2009; Zühlke et al., 2016), which was shown to most effectively disrupt the enormously robust cells of the spheric, Gram-positive CF key pathogen *S. aureus* followed by subsequent boiling of the samples in an harsh SDS-based extraction buffer with a final SDS concentration of 1% adapted from (Chourey et al., 2010). Using this method, we were able to extract the highest protein amounts out of the enriched microbial fraction ranging from approximately 40 to 110 µg, which is sufficient for subsequent metaproteome analysis.

Taken together, we established and optimized a sputum-processing protocol for microbial enrichment characterized by the following major steps (i) mechanical and enzymatic homogenization, (ii) differential centrifugation as the first microbial enrichment step, (iii) liquefaction with DTT, (iv) filtration as the second microbial enrichment step, (v) optimized cell disruption and protein extraction by a combination of beat beating and boiling in SDS extraction buffer.

#### Microbial Proteins Were Enriched by a Maximum Factor of 2.7

After we established a protocol for microbial enrichment, we selected three different patients, designated Patient A, Patient B, and Patient C, for detailed metaproteome analyses as a proof of concept. In order to assess the enrichment efficiency of these three sputum samples, we compared a non-enriched control and the enriched sample using a state-of-the-art metaproteomics workflow (**Figure 1**) and monitored microbial cell count microscopically (**Figure 2**). For metaproteome analyses, we measured two technical replicates of each sample in LC-MS/MS experiments showing decent reproducibility (**Figure S3**). However, the overall percentage of assigned spectra is rather low compared to other metaproteomics datasets (Hinzke et al., 2019), which most likely might be attributed to the typically high proteolysis rates within CF sputum caused by neutrophil-derived proteases (Sloane et al., 2005; Folkesson et al., 2012).

**Figure 3:**
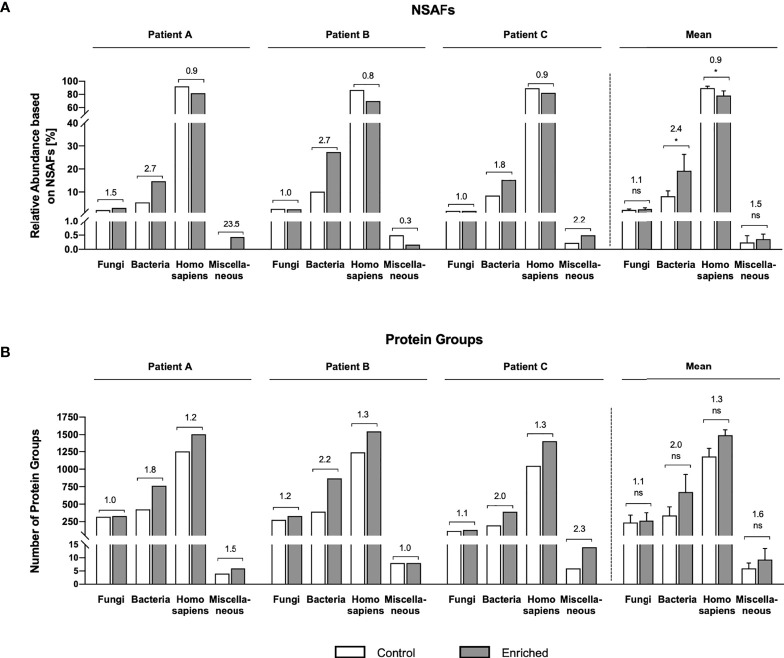
Microbial enrichment success based on metaproteome data. Abundance of fungi, bacteria, Homo sapiens, and Miscellaneous (containing viruses as well as non-assigned proteins) is expressed based on mean values of two technical replicates for each patient as **(A)** NSAF-based relative abundance and **(B)** absolute number of protein groups. Numbers above bars represent enrichment factors of proteins/protein groups after enrichment compared to the control. Data on the right side of the figure represent the mean values of Patient A–C ± standard deviation and indicate statistical significance after multiple unpaired t-tests; * = statistically significant (p < 0.05), ns, not significant.

Two different complementary read-outs were used to evaluate microbial protein enrichment efficiency: relative protein abundance based on NSAFs, and the number of identified proteins/protein groups (= group of proteins sharing the same identified peptide(s)) (**Figure 3**). Briefly, NSAF-based quantification of proteomic data refers to a label-free quantification method relying on a spectral counting approach. More precisely, quantification of proteins is carried out by comparing the number of identified MS/MS spectra of a specific protein over several LC-MS/MS experiments, since protein abundance correlates with the number of proteolytic peptides and thus with the number of total MS/MS spectra (spectral counts). Considering that large proteins naturally contribute a higher number of peptides/spectra compared to small proteins, spectral counts undergo normalization to create the NSAF. Therefore, the number of spectral counts (SC) of a specific protein is divided by the protein’s length (L), divided by the sum of all SC/L values from the given experiment (Zhu et al., 2010) allowing relative quantification of proteins throughout samples.

Both, the NSAF as well as the protein group-based evaluation showed a clear trend of successful enrichment of bacterial proteins and depletion of human proteins in all three samples (**Figure 3**). In fact, the 250 most prominent human proteins (e.g. including mucins, albumins, immunoglobulins), which contribute to the total proteome mass by approx. 40%, were depleted by a mean factor of 1.6 fold (**Tables S3**–**S5**). Regarding the enrichment of bacterial proteins, NSAF-based enrichment factors range from 1.8-fold (Patient C) to 2.7-fold (Patient A and Patient B) (**Figure 3A**). Notably, these enrichment factors are also well reflected by our microscopic analyses. 50 randomly selected fields of view were acquired for the different steps of the protocol. Both qualitatively (**Figure 2A**) as well as quantitatively (**Figure 2B**) we observed a clear reduction of human (epithelial) cells and particles bigger than 50 µm in our samples after the first step of differential centrifugation (**Figure 1**). Particles smaller than 10 µm as well as microbial cells were obviously enriched after filtration (**Figure 2**). Notably, the mean enrichment factor of bacterial cells calculated from microscopic analyses of 2.7-fold is very close to the bacterial enrichment factors calculated from NSAFs.

**Figure 4:**
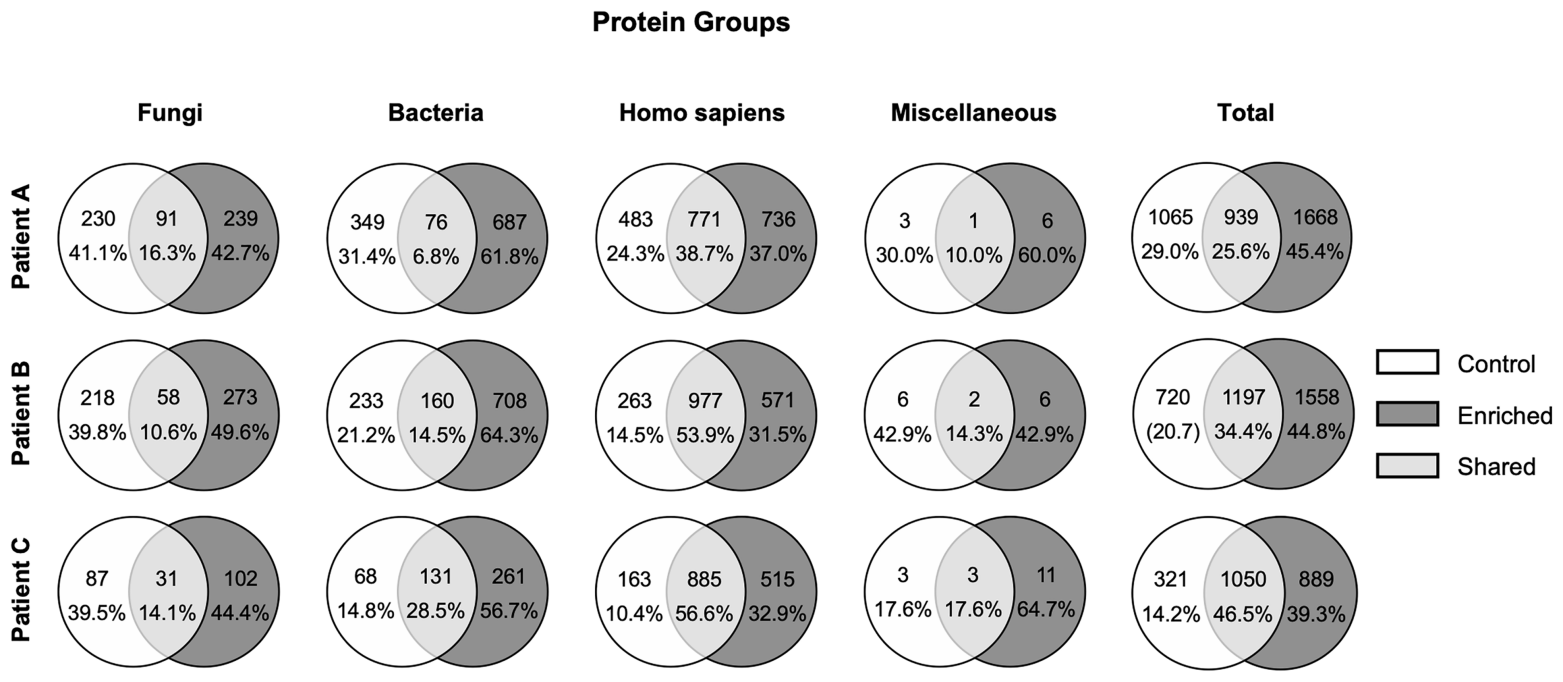
Venn diagrams visualizing absolute numbers and percentages of identified proteins/protein groups. Numbers of proteins/protein groups identified in control samples are depicted in white circles, proteins/protein groups identified in enriched samples are depicted in dark grey, and shared proteins/protein groups are depicted in light grey. According to taxonomic assignments by Prophane, proteins/protein groups of each sample are assigned to Fungi, Bacteria, Homo sapiens, and Miscellaneous (containing viruses as well as non-assigned proteins), respectively, and are summed up to total numbers.

Protein group-based enrichment factors for bacterial proteins range from 1.8-fold (Patient A: 425 total protein groups in control, 763 after enrichment), to 2.0 fold (Patient C: 199 total protein groups in control, 392 after enrichment), and 2.2-fold (Patient B: 393 total protein groups in control, 868 after enrichment) (**Figures 3B**, **4**). However, considering that the total number of identified proteins/protein groups is overall higher after enrichment compared to the control, the enrichment factors need to be normalized accordingly. Doing so, normalized protein group-based enrichment factors range from 1.4-fold (Patient A and C) to 1.5-fold (Patient B).

Total numbers of protein groups depicted in **Figure 4** indicate a rather small overlap between proteins/protein groups found in the control and the enriched fraction, respectively. This overlap ranges from 6.8% (Patient A), to 14.5% (Patient B) and 28.5% (Patient C). One explanation for this might be that a high proportion of bacterial proteins was lost during the enrichment process. Surprisingly, these potentially lost proteins are only partially annotated as extracellular proteins (e.g. nucleases and toxins, **Tables S3**–**S5**), which indicates that a great number of proteins in the extracellular sputum milieu are derived from cell lysis (e.g. proteins belonging to energy metabolism or DNA replication, ribosomal proteins, stress response proteins, **Tables S3**–**S5**).

#### Evaluation of the Bias Introduced by Microbial Enrichment

**Figure 5:**
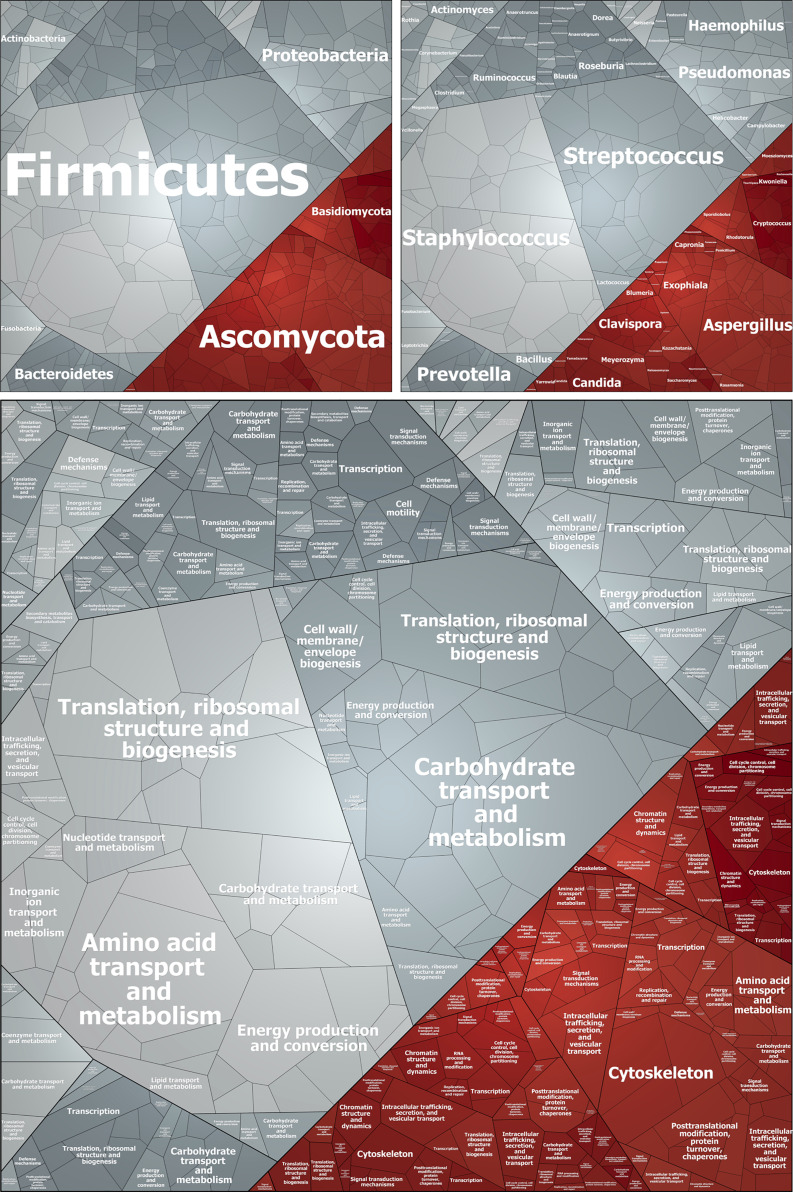
Voronoi treemap visualizing the taxonomic and functional affiliation of bacterial (grey) and fungal (red) protein/protein groups identified after enrichment in Patient A. Each cell represents a single protein/protein group, which size correlates with NSAF-based protein abundance. Proteins/protein groups are clustered according to Prophane results based on their taxonomic assignment on class level (upper left), genus level (upper right), and based on their functional assignment (lower panel). Proteins of unknown function are excluded from this visualization.

In general, every enrichment process introduces a bias. E.g. the composition of microbial proteins in stool changes in the ratio of Firmicutes- and Bacteriodetes-derived proteins after differential centrifugation and in the proportion of extracellular and host proteins (Tanca et al., 2015). This emphasizes the relevance of the chosen processing protocol influencing metaproteome data acquisition. Here, we cannot exclude that we lost big multicellular aggregates/biofilms during the enrichment. To address this problem, it might be useful to analyze different fractions during sputum processing in order to increase bacterial protein identification coverage. For instance, extracellular proteins can easily be enriched and extracted using Strata-Clean beads as described by (Bonn et al., 2014; Graf et al., 2019) (data not shown) and subsequently analyzed by metaproteomics. However, to exclude that a systematic error is inherent with our enrichment protocol and the abundancies of bacterial proteins are not excessively over- or underrepresenting any bacterial species, we compared the NSAF-based protein abundances of different species with CFU counts revealing a reasonable relation (**Table 1**, **Figure 5** and **Figures S4**, **S5**).

Interestingly, according to our metaproteome data, fungal cells/proteins were not enriched (**Figure 3**). This might be explained by the varying cells size of different fungal species in yeast or hyphae form e.g. ranging from 4 to 12 µm in diameter for yeast cells, 1 to 3 µm in diameter and several 100 µm in length for hyphae, and 1 to 5 µm for spores and conidia (Hickey and Read, 2009; Chotirmall and McElvaney, 2014; Thomson et al., 2016; Williams et al., 2016). This means, that small fungal cells would be enriched during our first enrichment step by differential centrifugation, but bigger fungal cells will be depleted during our second enrichment step by filtration (cut off 10 µm). However, those fungal genera, which are most frequently identified in CF in the literature, match the genera we identified as the most abundant by metaproteomics: namely, *Aspergillus* (prevalence up to 57%), *Candida*, *Blumeria*, *Exophilia*, *Clavispora*, and *Cryptococcus* (**Figure 5** and **Figures S4**, **S5**) (Chotirmall and McElvaney, 2014; Williams et al., 2016; Tracy and Moss, 2018). The genus *Scedosporium* (prevalence ranging from 3.1% to 10.6% (Williams et al., 2016), however, plays a minor role according to our data (**Figure 5** and **Figures S4** **1**, **S5**).

### Important Bacterial (Patho-)Physiological Pathways Revealed by Metaproteome and Metabolome Analyses

The total number of identified proteins/protein groups after enrichment differs from patient to patient. In more detail, we identified 2607 proteins/protein groups for Patient A, 2755 for Patient B and the lowest number of 1939 for Patient C (**Figure 4**) – the patient whose microbial lung community is dominated by *P. aeruginosa* and who shows the lowest lung function (**Table 1** and **Table S1**, **Figure S4**). Interestingly, these results are in line with the literature stating a exacerbation/reduced lung function due to *P. aeruginosa* infection, which is caused by the extensive recruitment of neutrophils and concomitant proteolytic digestion of lung tissue and proteins of bacterial pathogens (Sloane et al., 2005; Folkesson et al., 2012). This neutrophil-derived proteolytic digestion in consequence likely leads to a reduced protein identification coverage (and reduced percentage of assigned spectra) as observed in Patient C (**Figure 4** and **Figure S3**).

**Figure 6:**
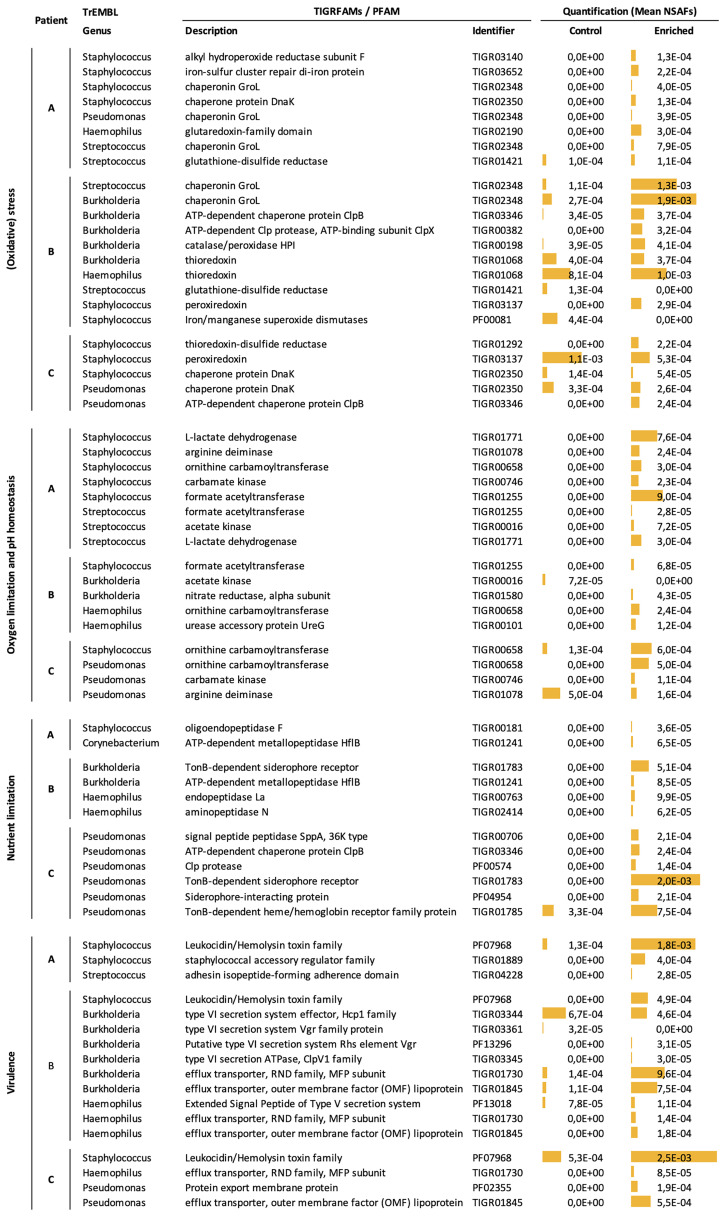
Selected Proteins/Protein Groups of important physiological pathways showing high abundances. Each row represents one protein/protein group assigned to (from left to right): a physiological pathway, a patient sample, a genus, a functional description, an identifier, a NSAF-based quantitative value (mean of two technical replicates) of the control and the enriched sample. Yellow bar graphs reflect NSAF values for an improved results visualization and eased results extraction for the reader.

The number of protein/protein groups assigned to the most prominent bacterial genera in CF like *Pseudomonas* (166 in Patient C), *Staphylococcus* (185 in Patient A), *Burkholderia* (408 in Patient B), *Haemophilus* (127 in Patient B), and *Streptococcus* (128 in Patient A) give a first insight into the physiology of these pathogens during CF infection. This means that there is still room for improvement of our enrichment protocol to ultimately increase bacterial protein identification coverage. Notably, the majority of protein groups assigned to the aforementioned dominant bacterial pathogens are poorly or even uncharacterized, indicating that important host-adaptation strategies of the identified pathogens have so far not been addressed and uncovered experimentally. Protein groups of known function identify various physiological pathways and virulence factors, which are key for bacterial pathogens to establish chronic infections: host immune evasion, anaerobic metabolism, and virulence/antibiotic resistance (Folkesson et al., 2012). We would like to emphasize that out of the 69 proteins/protein groups related to the above mentioned traits (**Figure 6**) 46 proteins/protein groups were exclusively identified in the enriched samples. Only 4 proteins/protein groups were exclusively identified in the control samples, while 19 proteins/protein groups were shared by control and enriched samples. Out of those 19 proteins/protein groups two were slightly less abundant in the enriched samples. Together this underlines the value of our enrichment protocol.

#### Oxidative Stress

During host immune response, large numbers of neutrophils are recruited, which fight pathogens by the production of reactive oxygen species (Folkesson et al., 2012; Kamath et al., 2015). Consistently, in Patient A we detected an alkyl hydroperoxide reductase (*Staphylococcus* sp.), a Glutathione S-transferase (*Haemophilus* sp.), an iron-sulfur-cluster repair protein (*Staphylococcus* sp.), glutathione-disulfide reductase (*Streptococcus* sp.), and the molecular chaperones DnaK (*Staphylococcus* sp.) and GroL (various species) as well as the protease ClpP (*Streptococcus* sp.), which all are involved in protein protection, repair, or degradation of proteins and inducible after various stress conditions like oxidative stress (Michta et al., 2014; Ezraty et al., 2017). Moreover, in Patient B *Burkholderia* sp. express the chaperone GroL, the protease ClpB and ClpX as well as catalase/peroxidase. In this sample, we additionally identified thioredoxin, glutathione disulfide reductase, peroxiredoxin, and iron/manganese superoxide dismutase expressed by *Burkholderia* sp., *Haemophilus* sp., *Streptococcus* sp., and *Staphylococcus* sp., respectively. In Patient C, we identified the following (oxidative) stress proteins: thioredoxin, peroxiredoxin, thioredoxin-disulfide reductase, DnaK, and ClpB expressed by *Pseudomonas* sp. and *Staphylococcus* sp. (**Tables S3**–**S5**). Collectively, these data suggest that the most important CF pathogens within our samples might cope with oxidative stress during CF infection, which is in line with the literature (Treffon et al., 2018).

#### Oxygen Limitation and pH Homeostasis

The lung environment and especially the CF lung is not considered to be entirely aerobic, due to the viscous character of mucus, oxygen consumption by colonizing microbes, and phagocytes. Rather, oxygen gradients ranging from hypoxic to even anoxic/anaerobic microenvironments characterize the CF-lung (Worlitzsch et al., 2002). Thus, even strict anaerobic bacteria are able to thrive in lungs of CF patients (Filkins and O'Toole, 2015). Consistent with hypoxic and anaerobic conditions, we identified marker-proteins of fermentative metabolism from *Staphylococcus* sp. including lactate dehydrogenase, formate acetyltransferase and acetate kinase in all three samples. In the samples of Patient A and Patient B we further identified formate acetyltransferase, L-lactate dehydrogenase and acetate kinase, assigned to the genera *Streptococcus, Staphylococcus*, and *Burkholderia*, respectively (**Figure 6** and **Tables S3**–**S5**). A further metabolic strategy to overcome oxygen limitation conserved in many pathogens is the fermentation of arginine *via* the arginine deiminase pathway (Lindgren et al., 2014). According to our metaproteome data, arginine deiminase, ornithine carbamoyltransferase, and carbamate kinase, are highly abundant and identified in multiple genera: *Staphylococcus* in Patient A and Patient C, *Haemophilus* in Patient B, and *Pseudomonas* in Patient C (**Figure 6** and **Tables S3**–**S5**). In support of our metaproteome data, we identified ornithine (Patient A-C) and citrulline (Patient B), key metabolites of the arginine deiminase pathway, by metabolic footprint analysis (**Figure S6**) (Lindgren et al., 2014). Clear induction of the arginine deiminase pathway suggests that not only ATP production, but also raise in pH due to production of ammonia is important for the pathogen to counteract acidification upon fermentation and likely upon phagocytosis by immune cells (Li et al., 2000; Beenken et al., 2004; Resch et al., 2005; Filkins and O'Toole, 2015). As evidence for anaerobic metabolism and acidification, we identified lactic acid as a fermentation product in all three samples (**Figure S6**). Moreover, we identified the urease accessory protein UreG from *Haemophilus* sp. in Patient B, which is a protein of the urease pathway and similarly to the arginine pathway is involved in pH homeostasis (**Figure 6** and **Table S4**) (Li et al., 2000). Notably, urea as the substrate of the urease pathway was also detected in Patient A and Patient B (**Figure S6**). Finally, we identified the alpha subunit of the nitrate reductase from *Burkholderia* sp. in Patient B as part of the nitrate respiration pathway (**Figure 6** and **Table S4**) (Filkins and O'Toole, 2015) utilizing nitrate as an alternative terminal electron acceptor.

#### Nutrient Limitation

The competition for nutrients within the CF airways is an important selective pressure influencing the composition of the CF community. For example Pseudomonads and Streptococci are able to efficiently utilize amino acids, organic acids and alcohols (partly produced by other community members) leading to high growth rates within the lung (Yang et al., 2008; Henson et al., 2019; La Rosa and Molin, 2019). Notably, our metabolome data revealed various amino acids (and organic acids) within all three sputum samples (**Figure S6**),which supports that amino acids are the major carbon- and nitrogen source in CF sputum (Palmer et al., 2005; La Rosa and Molin, 2019). Moreover, we found evidence that these amino acids could result from the hydrolytic activity of a variety of different proteases and peptidases (Kamath et al., 2015; Quinn et al., 2019). Currently, proteases of human origin are believed to be the key players responsible for proteolytic digestion in the CF airways. Specifically, human neutrophil-derived elastase and cathepsins are considered to be the most abundant and potent proteases (Voynow et al., 2008). Although we identified these human proteases among the most abundant in our proteome data, we additionally found a great number of proteases and peptidases of bacterial origin. For example, we identified a staphylococcal oligopeptidase in Patient A, the metallopeptidase HflB, peptidase Do, and protease HslVU from *Burkholderia* sp. and *Streptococcus* sp. as well as endopeptidase La, and aminopeptidase N from *Haemophilus* sp. in Patient B, and multiple Clp protease proteins in Patient C (**Figure 6** and **Table S3**–**S5**). In fact, by using NSAFs to calculate the contribution of all human and all bacterial proteases and peptidases to the total proteome mass, we found that the human proteases/peptidases in the non-enriched control accounts for 2.15% of the total proteome mass and bacterial proteases/peptidases in the enriched sample for 0.84% (**Figure 6** and **Tables S3**–**S5**). This suggests that the role of bacterial proteases and peptidases in the CF pathophysiology is larger than previously acknowledged (Kamath et al., 2015).

Another vital nutrient during CF infection is iron, which is needed by bacteria as a cofactor in essential metabolic enzymes (Reid et al., 2009). Although the iron concentration within the CF airway is relatively high compared to other human body sites, pathogens within the CF lung fight for iron by sequestering iron chelating siderophores and proteases degrading transferrin, lactoferrin, and heme-containing proteins like hemoglobin and myoglobin (Reid et al., 2009; Runyen-Janecky, 2013; Treffon et al., 2018). This fight for iron is well reflected by our metaproteome data, e.g. revealing multiple TonB-dependent siderophore receptors and TonB-dependent heme/hemoglobin receptor family proteins of *Burkholderia* sp. and *Pseudomonas* sp. in Patient B and Patient C, respectively (**Figure 6** and **Tables S3**–**S5**).

#### Virulence Factors

Among the identified microbial proteins many important virulence factors were detected. For instance, staphylococcal leukocidin, an immune evasion protein that mediates lysis of leukocytes (Scherr et al., 2015) was very abundant in all three samples, underlining the ongoing battle between the host immune system and the pathogen within the CF-lung (**Figure 6** and **Tables S3**–**S5**). However, our proteomic data did not provide support for presence of microbial biofilms in the samples, which is considered a major microbial phenotype during infection (Folkesson et al., 2012; Filkins and O'Toole, 2015; Kovach et al., 2017). Only a streptococcal adhesin (Patient A), one adhesin of *Pseudomonas* sp. (Patient C), and the aforementioned staphylococcal leukocidins (Patient A-C), which have the potential to moonlight as a stabilizing extracellular matrix component under acidic conditions, were detected (**Figure 6** and **Tables S3**–**S5**) (Graf et al., 2019). We cannot entirely exclude that the lack of biofilm-related proteins in our sample is related to the enrichment procedure, as large cellular clusters were cleared from the sample during the first centrifugation and/or the filtration step.

Another well described concept of bacterial virulence are secretion systems, which transport effectors or DNA across membranes to manipulate the physiology of host cells or competing bacteria (Voth et al., 2012). Indeed, we identified multiple proteins of a Type VI secretion system of *Burkholderia* sp. in Patient B. Furthermore, we found an Extended Signal Peptide of Type V secretion system protein in *Haemophilus* sp. (Patient B) (**Figure 6** and **Tables S3**–**S5**). This emphasizes the importance of secretion systems, especially for *Burkholderia* sp., during successful CF infection. Finally, we found multiple efflux transporter mediating antibiotic resistance (Li and Nikaido, 2009) in *Burkholderia* sp., *Haemophilus* sp., *Pseudomonas* sp. (Patient B and Patient C), which likely reflect a response towards the antimicrobial therapy that the three investigated patients underwent (**Figure 6**, **Table 1** and **Tables S4**, **S5**).

### Limitations of Our Study

The detailed analysis of three sputum samples provided novel insights into the microbial pathophysiology within the CF lung and revealed high expression of the arginine deiminase pathway and multiple proteases, demonstrating the applicability of our protocol. We are aware that larger sample numbers will be required to validate the significance of these findings. Future studies should not only include larger sample numbers but should also consider specific mutations of the CFTR gene and the individual patient treatment regimens to account for the high in-between variability of CF patients (Tanca et al., 2014). Additionally, larger numbers of biological and technical replicates will also increase protein coverage. Including a proteomic analysis of the supernatant of the differential centrifugation step II could provide further insight into the microbial secretome (Bonn et al., 2014; Graf et al., 2019) (**Figure 1**). An additional question that deserves further investigation is the small overlap of protein groups between enriched and non-enriched samples (**Figure 4**) (Starr et al., 2017). Moreover, to increase the coverage of our metabolome analysis (La Rosa and Molin, 2019), in addition to PBS-buffer based metabolite extraction combined with GC-MS analyses resulting in the identification of 52 metabolites, further metabolite extraction and analysis methods e.g. according to (Yang et al., 2012; Quinn et al., 2015) could be beneficial. Finally, measuring absolute metabolite concentrations will improve the comparison of metabolite levels between different studies.

## Conclusions

We established an innovative, reliable, and easy-to-handle sputum processing protocol for *in vivo* metaproteome analyses. With this protocol in hand, we provide the first *in vivo* study of microbial CF sputum communities combining metaproteomic and metabolomic analyses supported by 16S sequencing and microscopic data as a proof of concept. Our metaproteome data show that we were able to enrich bacterial proteins by a maximum factor of 2.7, thereby increasing protein identification coverage to a level, which provides novel valuable insights into bacterial CF-lung pathophysiology. Our early data, which are derived from just 3 sputum samples proof the applicability of our protocol but do lack statistical power. However, they still indicate that the infecting bacteria might be coping with oxygen and nutrient limitation as well as oxidative stress and the human immune system, respectively. Our early data also provide evidence that the arginine deiminase pathway as well as bacterial proteases play an underappreciated role in CF pathophysiology.

## Data Availability Statement

The original contributions presented in the study are publicly available in NCBI (using accession number PRJNA741386 for 16S sequencing data) and within the ProteomeXchange consortium *via* the PRIDE partner repository (using dataset identifier PXD025134 for metaproteome data).

## Ethics Statement

The studies involving human participants were reviewed and approved by Institutional ethics review board Münster, Germany (2010-155-f-S). The patients/participants provided their written informed consent to participate in this study.

## Author Contributions

AG, JP-F, and KR were responsible for the study conceptualization. BK carried out the prospective study including CF sputum sampling, collection of clinical data and microbiological work-up of sputum specimens. AG and JS performed the experiments to develop the sputum processing protocol. 16S sequencing analyses were performed by DP. AG, TS, and DB performed metaproteome analyses and ML and MW were responsible for metabolome analyses. DP analyzed 16S sequencing data, while AG analyzed metaproteome and metabolome data. AG, JP-F and KR wrote the manuscript, which was critically edited by all other co-authors. All authors contributed to the article and approved the submitted version.

## Funding

This work was funded by the German research foundation (https://www.dfg.de/en/) Collaborative Research Center Transregio 34, subprojects A3 to KR, A8 to JP-F, C7 to BK, Z2 to DB, and Z4 to ML. The funders had no role in study design, data collection and analysis, decision to publish, or preparation of the manuscript.

## Conflict of Interest

The authors declare that the research was conducted in the absence of any commercial or financial relationships that could be construed as a potential conflict of interest.

## Publisher’s Note

All claims expressed in this article are solely those of the authors and do not necessarily represent those of their affiliated organizations, or those of the publisher, the editors and the reviewers. Any product that may be evaluated in this article, or claim that may be made by its manufacturer, is not guaranteed or endorsed by the publisher.
