# Supplementary material for: An Innovative Protocol for Metaproteomic Analyses of Microbial Pathogens in Cystic Fibrosis Sputum
Source: Front Cell Infect Microbiol. 2021 Aug 27;11:724569. doi: 10.3389/fcimb.2021.724569 (PMC8432295; doi:10.3389/fcimb.2021.724569)
Supplement: Supplementary file 1 [file DataSheet_1.pdf]

Supplemental Figure 1

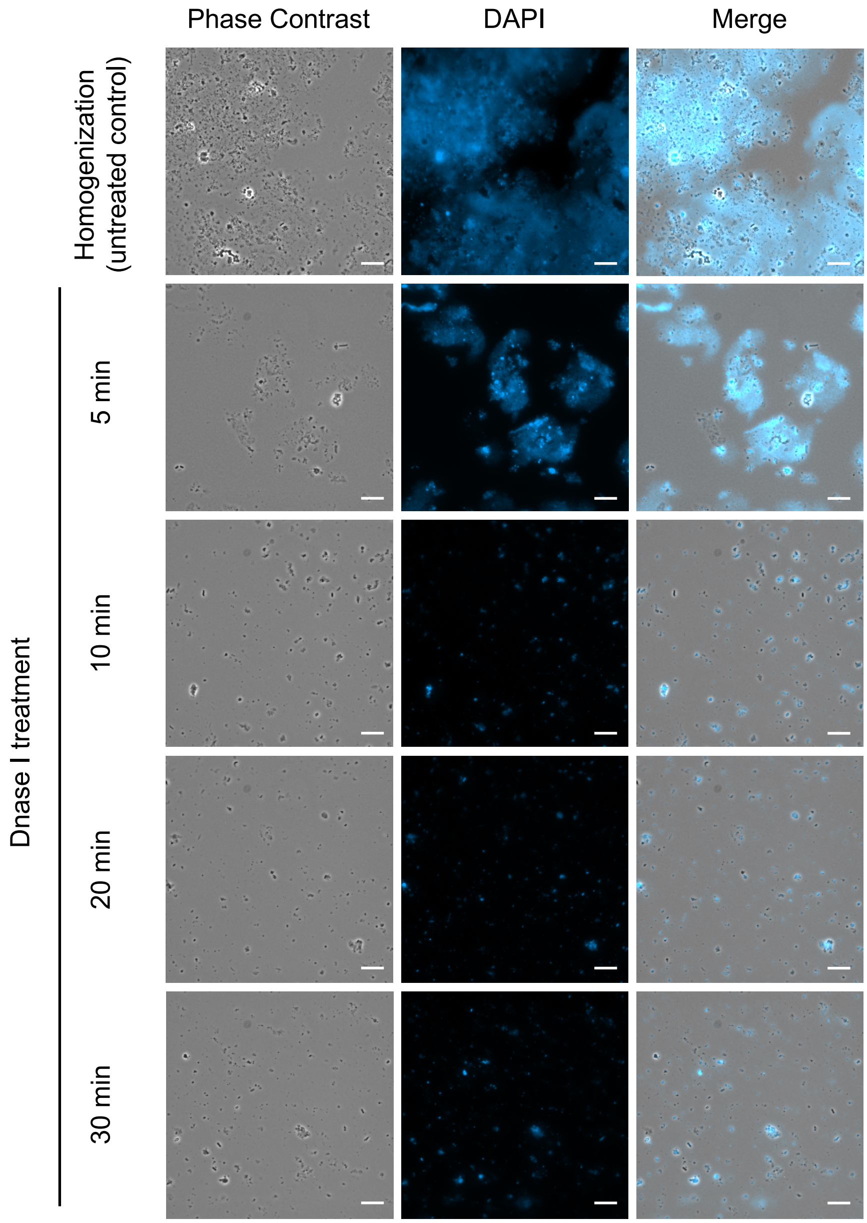

Fig. S1: Microscopic evaluation of DNase I treatment times. Sputum samples after gross homogenization (non-treated control) were treated with DNase I for different time periods and digestion efficiency was qualitatively evaluated. Representative images are show. Bar graphs represent 10 µm.
