# Supplementary material for: An Innovative Protocol for Metaproteomic Analyses of Microbial Pathogens in Cystic Fibrosis Sputum
Source: Front Cell Infect Microbiol. 2021 Aug 27;11:724569. doi: 10.3389/fcimb.2021.724569 (PMC8432295; doi:10.3389/fcimb.2021.724569)
Supplement: Supplementary file 2 [file DataSheet_2.pdf]

Supplemental Figure 2

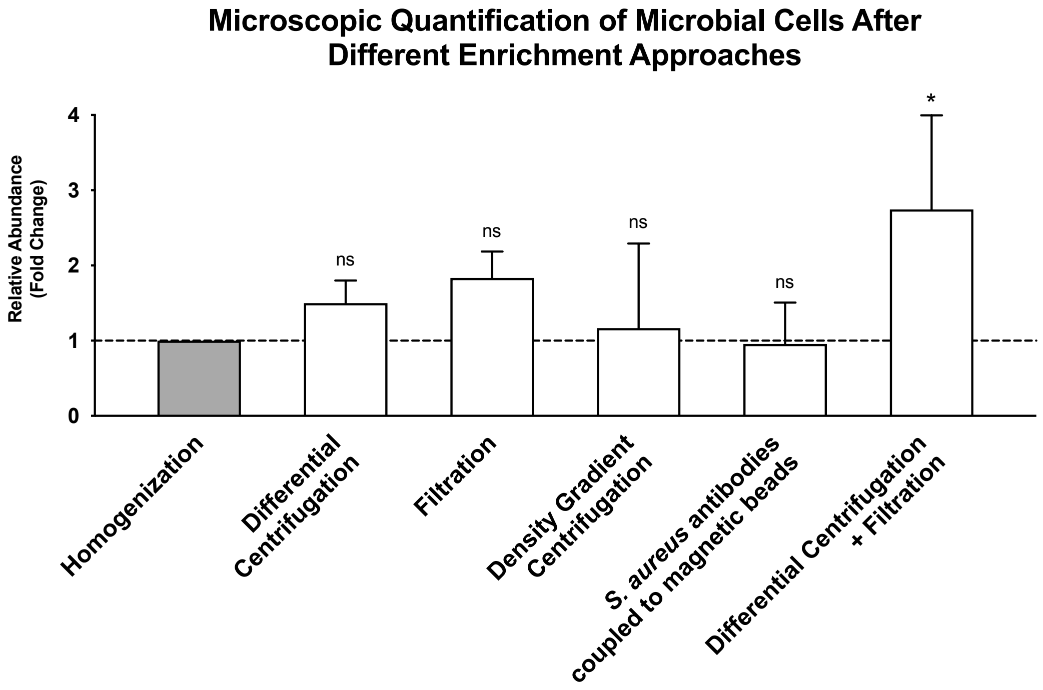

Fig. S2: Microscopic Quantification of Microbial Cells after Different Enrichment Approaches. Microbial Cells were counted in 50 randomly selected fields of view and mean values were calculated. The number of microbial cells in the homogenized control (grey bar) was set to 1 and relative amounts of microbial cells were depicted as fold changes in relation to the homogenized control ± standard deviation. Statistical significance is indicated after multiple unpaired t-tests (* = statistically significant ($p<0.05$), ns = not significant). For the enrichment approach of *S. aureus* cells using antibodies coupled to magnetic beads only the number of spheric cocci of approximately 1 mm diameter were counted.
