# Supplementary material for: An Innovative Protocol for Metaproteomic Analyses of Microbial Pathogens in Cystic Fibrosis Sputum
Source: Front Cell Infect Microbiol. 2021 Aug 27;11:724569. doi: 10.3389/fcimb.2021.724569 (PMC8432295; doi:10.3389/fcimb.2021.724569)
Supplement: Supplementary file 3 [file DataSheet_3.pdf]

Supplemental Figure 3

A

Patient	Sample	Technical Replicate	Acquired spectra	Assigned Spectra	% Assigned Spectra	Correlation Coefficient Squared (R^2)
A	Control	I	248710	26846	10.8	0.85
		II	272477	24303	8.9	
	Enriched	I	263018	30581	11.6	0.99
		II	194892	23355	12.0	
B	Control	I	346101	43521	12.6	0.93
		II	343589	40586	11.8	
	Enriched	I	305429	43915	14.4	0.90
		II	297356	42362	14.2	
C	Control	I	378847	25762	6.8	0.90
		II	385258	24080	6.3	
	Enriched	I	347108	29593	8.5	0.91
		II	354596	29493	8.3	

B

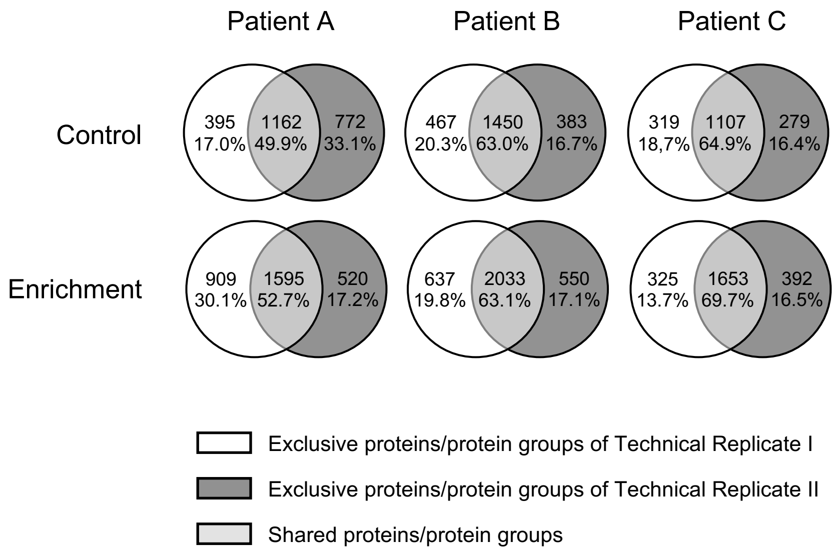

Fig. S3: Reproducibility of the two MS/MS runs (Technical Replicate I and II, respectively) for metaproteome analysis of each control and enriched sample. (A) Total number of acquired spectra, assigned spectra, and percentage of assigned spectra based on Scaffold Protein Reports as well as the correlation coefficient squared (R^2) obtained after comparing NSAFs of both replicates. **(B)** Exclusively identified proteins/proteins groups of Technical Replicate I are depicted in white, exclusively identified proteins/proteins groups of Technical Replicate II are depicted in dark grey, shared proteins/protein groups are depicted in light grey.
