# Supplementary material for: An Innovative Protocol for Metaproteomic Analyses of Microbial Pathogens in Cystic Fibrosis Sputum
Source: Front Cell Infect Microbiol. 2021 Aug 27;11:724569. doi: 10.3389/fcimb.2021.724569 (PMC8432295; doi:10.3389/fcimb.2021.724569)
Supplement: Supplementary file 4 [file DataSheet_4.pdf]

Supplemental Figure 4

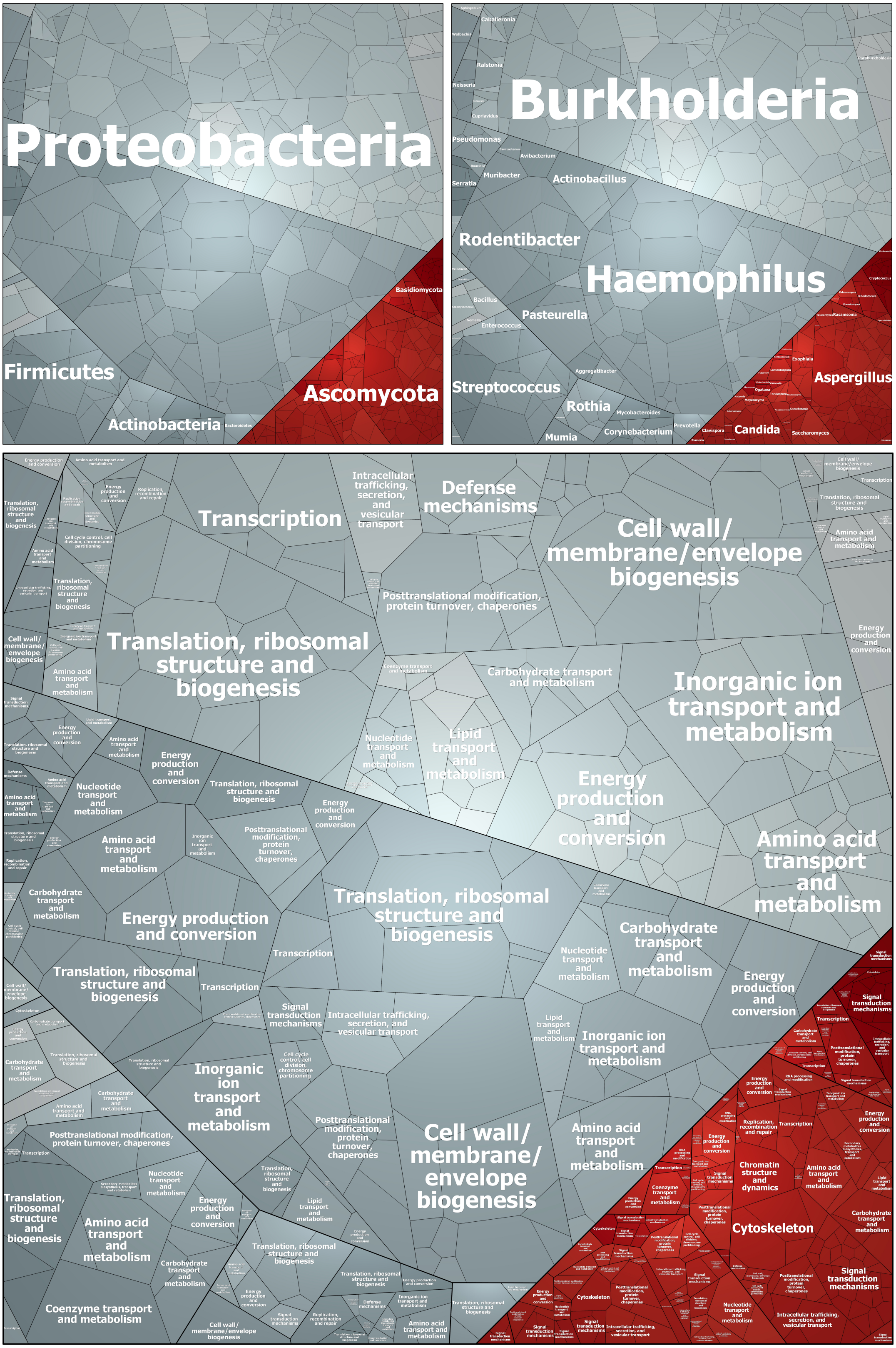

Fig. S4: Voronoi treemap visualizing the taxonomic and functional affiliation of bacterial (grey) and fungal (red) protein/protein groups identified after enrichment in Patient B. Each cell represents a single protein/protein group, which size correlates with NSAF-based protein abundance. Proteins/protein groups are clustered according to Prophane results based on their taxonomic assignment on class level **(upper left)**, genus level **(upper right)**, and based on their functional assignment **(lower panel)**. Proteins of unknown function are excluded from this visualization.
