# Supplementary material for: An Innovative Protocol for Metaproteomic Analyses of Microbial Pathogens in Cystic Fibrosis Sputum
Source: Front Cell Infect Microbiol. 2021 Aug 27;11:724569. doi: 10.3389/fcimb.2021.724569 (PMC8432295; doi:10.3389/fcimb.2021.724569)
Supplement: Supplementary file 6 [file DataSheet_6.pdf]

Supplemental Figure 6

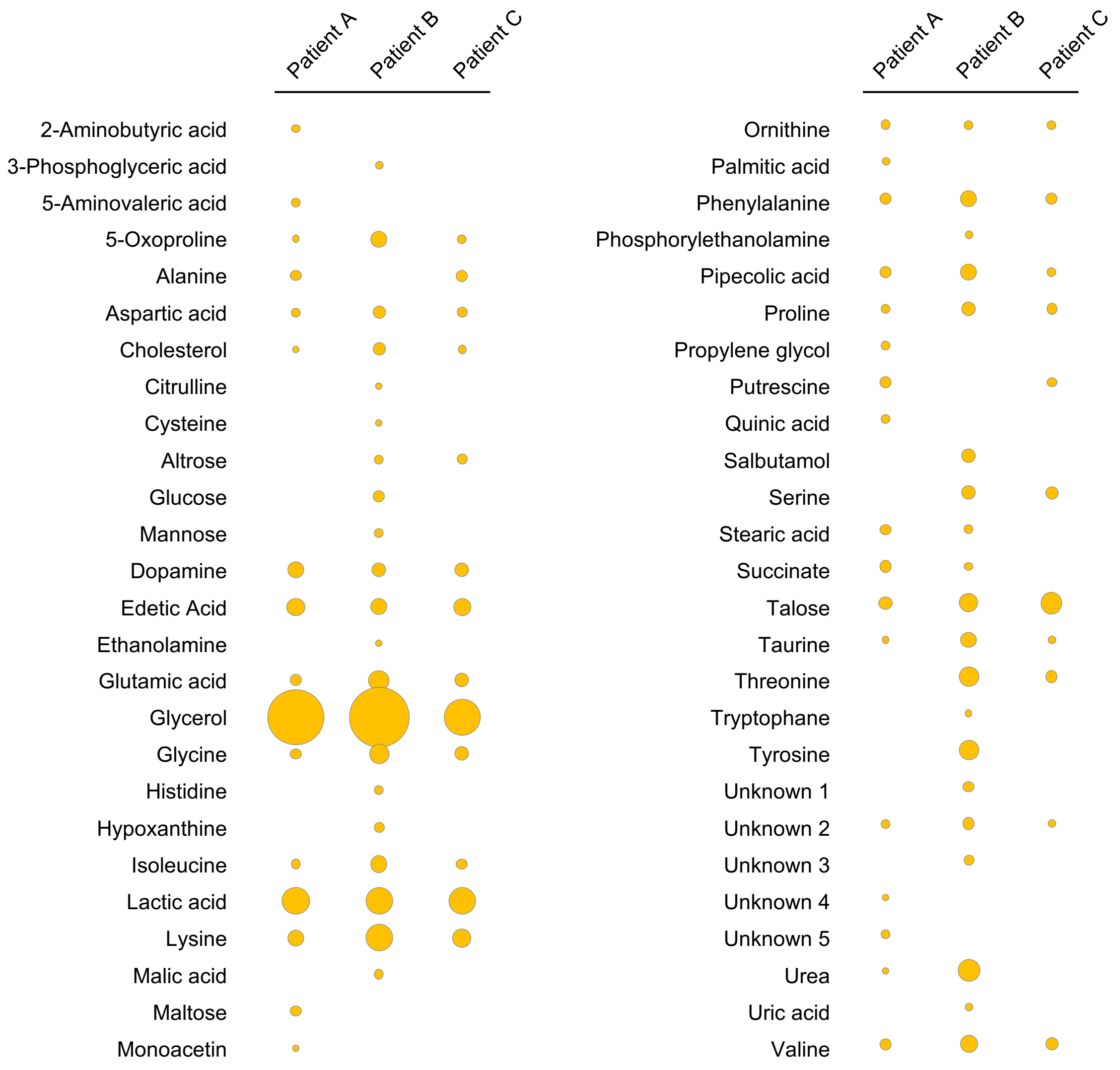

Fig S6.: Metabolic footprint analyses of CF sputum samples. Metabolites were extracted in PBS and analyzed by GC-MS**.** Relative metabolite concentrations are depicted as circles, which areas correlate with metabolite abundance.
