# Supplementary material for: An Innovative Protocol for Metaproteomic Analyses of Microbial Pathogens in Cystic Fibrosis Sputum
Source: Front Cell Infect Microbiol. 2021 Aug 27;11:724569. doi: 10.3389/fcimb.2021.724569 (PMC8432295; doi:10.3389/fcimb.2021.724569)
Supplement: Supplementary file 7 [file DataSheet_7.pdf]

Supplemental Table 2:Information about Prophane Settings

```
a. general information
   job name: Sample A
   job comment:
   start time: 2019-03-14 21:15:41
   end time: 2019-03-14 21:15:47

b. program versions
   prophane: 3.1.4

c. input
   protein report (Scaffold): 190201_ProteinReport_C1.xls
   report style: scaffold_4_8_x.yaml
   sequence data:
      - 181029_Sputum-DB_A_Linux_NR.fasta
   taxonomic data: None

d. sample groups
   1
      - ::A_TR1_1
      - ::A_TR2_1
   3
      - ::A_TR1_3
      - ::A_TR2_3

e. tasks
   task: 0
   task comment: tax_from_trembl_20180808_qcover90
   algorithm: diamond blastp
   query: sequences without taxonomic classification
(missing_taxa.faa)
   database: Uniprot Trembl database
   database version: 2018-08-08
   database comment: downloaded at 2018-08-08
   database scope: tax
   parameter:
      - evalue 0.01
      - query-cover 0.9
      - max-target-seqs 1

   task: 1
   task comment: fun_from_TIGRFAMs_15_cut_tc
   algorithm: hmmscan
   query: all sequences (all.faa)
   database: Tigrfams
   database version: 15.0
   database comment: downloaded at 2018-07-29
   database scope: func
   parameter:
      - evalue  0.01
      - cut  cut_tc
```

task: 2
task comment: fun_from_PFAMs_31_cut_ga
algorithm: hmmscan
query: all sequences (all.faa)
database: Pfam-A
database version: 31
database comment: downloaded at 2018-07-29
database scope: func
parameter:
- evalue 0.01
- cut cut_tc

task: 3
task comment: fun_from_eggNog_4.5.1
algorithm: emapper
query: all sequences (all.faa)
database: eggmap database
database version: 4.5.1
database comment: includes euk, viruses, bakt, arch; downloaded at 2018-07-31
database scope: func
parameter:
- m diamond
